# The Good (Tumor Killing) and the Bad (Cardiovascular Complications) of Immunologic Checkpoint Inhibitors

**DOI:** 10.1007/s11886-024-02147-x

**Published:** 2024-10-23

**Authors:** Maria T. Gamero, Avish Patel, Eugene Storozynsky

**Affiliations:** Department of Medicine, Division of Cardiovascular Disease, Jefferson Heart Institute, Thomas Jefferson University Hospital, Philadelphia, PA USA

**Keywords:** ICI Immune checkpoint inhibitor, IrAE immune related adverse events, CTLA 4 cytotoxic T lymphocyte associated protein 4, PD 1 programmed cell death protein 1, PD L1 programmed death ligand 1

## Abstract

**Purpose of Review:**

This review details the significant advancement in knowledge of Immune-checkpoint inhibitor (ICI) and its potential deleterious cardiac immune-related adverse effects (irAE). We explore their mechanisms on the cardiac tissue, providing guidance on risk factors, clinical presentations, diagnostic strategies along with treatment.

**Recent Findings:**

Recent findings have provided insights of cardiac irAEs that exist beyond the previously well-known ICI-induced myocarditis. We have a better understanding of the wide variety of cardiac irAEs pathologies both early and late onset. Moreover, there is more data on mechanisms of cardiotoxicity and patient and therapy-related risk factors, supporting closer routine cardiac monitoring with biomarkers and imaging for prevention and early detection.

**Summary:**

Diagnosing cardiac irAEs is a challenge given its broad clinical presentation. A high-level of suspicion in addition to early work-up is crucial to prevent serious cardiac events. A multi-disciplinary team including Cardiologists and Oncologists is essential for closely monitor patients’ cardiac status on ICI therapy. There is a need of updated guidelines to establish clear recommendations in patients on ICIs.

## Introduction

### Definition of ICI

Immune-checkpoint inhibitors (ICI) are a novel type of anti-tumor therapy. This is a class of medications demonstrated to treat tumors with much improved outcomes with increased life expectancy and decreasing deaths [1]. They truly represent a remarkable advancement in treatment of malignancy by altering our own immune system to enhance anti-tumor responses. Tumor cells have the ability to decrease our own innate immune system’s ability to fight against the tumor cell itself. ICI’s specifically target these inhibiting pathways that cause the dampened response against tumor cells. This in turn should enhance the immune response of the body by allowing it to respond to triggers as it normally would even in the presence of tumor cells. These triggers can include infections for example [2].

The novel advancement with heightened results in management of malignancy does not come without a cost, especially when pathways in our immune system are involved as the target. Although many of the immune related adverse events (irAE) are minor with effects on the cardiac, pulmonary, gastrointestinal, nervous, hematologic, ocular, and skin systems, the events that are serious can be fatal. This emphasizes the need to bring awareness to adverse events from ICI therapy, how they can progress, and consequences if missed or not screened for properly. One of the most fatal are cardiac irAE which in of themselves are very rare [1].

### Mechanism of T Lymphocyte Activation

T lymphocyte activation is a well understood cascade that requires awareness to appreciate the mechanism of ICI’s and their impact on the human body from fighting malignancy to the potential adverse effects that can occur as will be discussed in this review. T lymphocytes play the most important role in the adaptive immune response. This response includes many receptors, attached molecules, and signaling cascades to function effectively. This also allows many targets to either inhibit or enhance this signaling cascade for which both tumor cells and anti-tumor therapies can use to target.

**Fig. 1:**
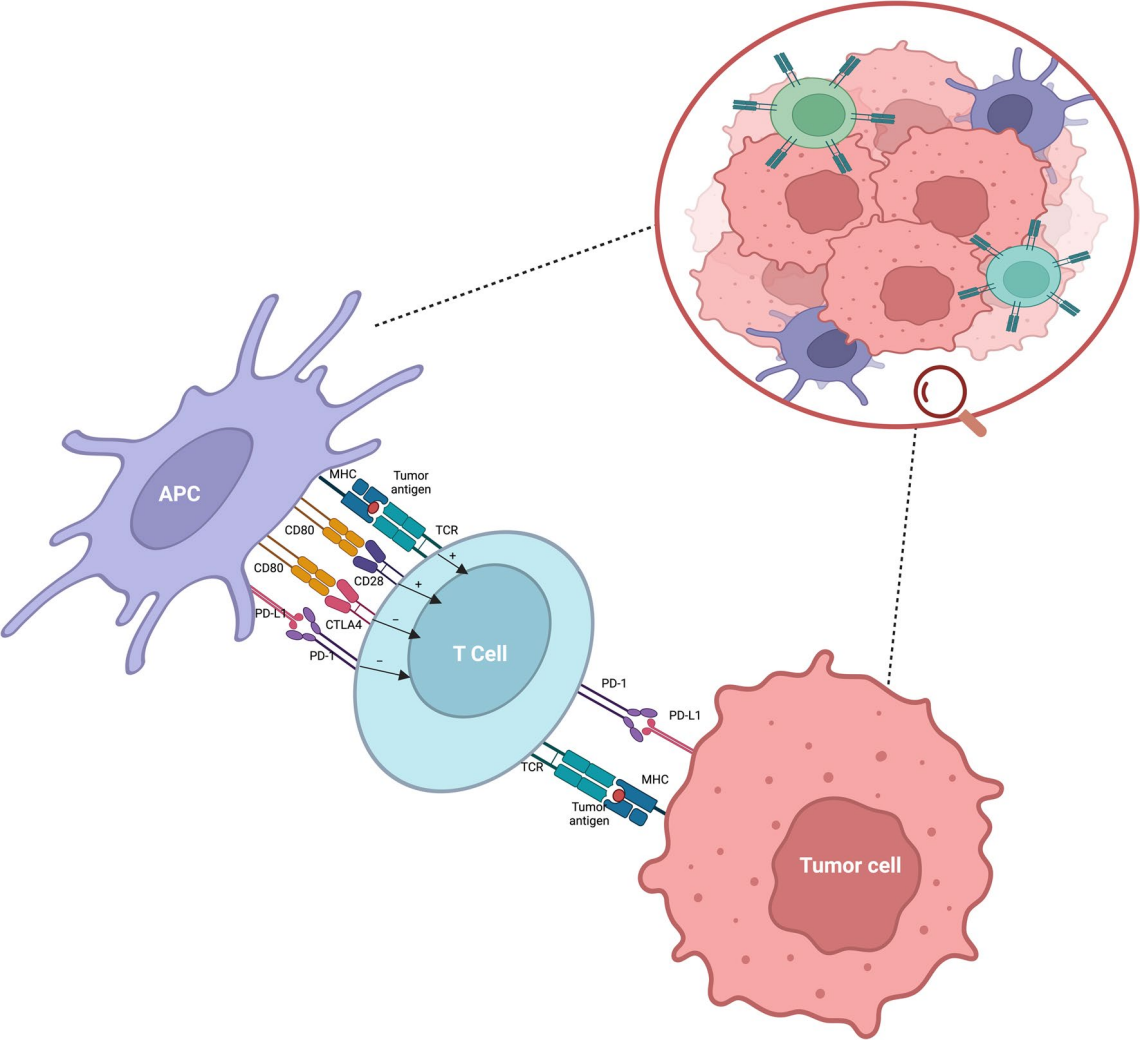
T cell activation and inhibitory receptor-ligand interactions involving TCR/MHC class I/II, CD28/CD80, CTLA-4/CD80, and PD-1/PD-L1. Key: APC: Antigen-presenting cell; PD-1: programmed cell death protein 1; PD-L1: Programmed death-ligand 1; TCR/MHC: T cell receptor/ Major histocompatibility complex class; CTLA-4: cytotoxic T-lymphocyte-associated protein 4

T lymphocytes first need to bind to antigen presenting cells (APC). The T cells have a receptor that is attached to the CD3 complex which binds directly to the anti-MHC complex on APC’s. This interaction is further stabilized with a co-stimulator binding of CD28 on T cells to B7 (CD80/CD86) molecules on APC’s which is needed for the activation of T cells to lead to the next step which is intracellular signaling (Fig. 1). Downstream signaling inside T cells includes molecules such as inositol 1,4,5-triphosphate (IP3) which releases intracellular calcium needed for promotion of transcription factors. Another molecule crucial is diacylglycerol which enhances the intracellular pathway bind activating protein kinase C. Another pathway involved leading to transcription of genes is the Ras and MAPK pathways [2]. All of these pathways and signaling cascades work together for T-cell activation leading to proliferation and differentiation of the appropriate T lymphocyte cell to function and release applicable molecules such as interleukins to mount a response to triggers in the body such as for infection or inflammation [3].

### Mechanism of Action of ICI

Tumor cells have the ability to bind immune checkpoint proteins with their own ligands. This in effect prevents the normal T cell mediated immune response to fight tumor cells. The ligands of tumor cells bind to checkpoint proteins such as cytotoxic T-lymphocyte-associated protein 4 (CTLA-4), programmed cell death protein 1 (PD-1), and lymphocyte activation gene-3 (LAG-3). These checkpoint proteins are expressed on T cell lymphocytes and are crucial to the signaling cascade that gives T lymphocytes their immune function. The tumor cell itself will bind to these checkpoint proteins on T cells and therefore inhibit the ability of the T cell effectively function. For example, T-cells have the checkpoint protein PD-1 which tumor cells bind to with their ligands PD-L1 and PD-L2. This causes the concept of T-cell exhaustion leading to lack of response by T-cells due to the inhibitor impact of this tumor to T cell binding [4–6]. This benefits the tumor cell as now the T cells in the body cannot mount a response to the tumor. The tumor to T cell binding causes an inhibitory signaling cascade within the T cell. It also in a way dampens the entire immune response in the body making the same person at risk of infections in addition to growth of the malignancy itself. Given the specificity of the tumor cells ability to bind specific proteins on T cells, the ICI are targeted to inhibit this binding. ICIs are medicines known as monoclonal antibodies. The mechanism of action of the ICIs involves the ICI binding to the checkpoint protein receptor itself on the T cell therefore blocking the ability of the tumor cell ligands to bind to the same T cell checkpoint inhibitor. This would in turn release any inhibitory signaling potential caused by tumor cells on T cells. This allows the T cells to function as normal without inhibition and can therefore recognize the tumor cells as foreign as they would in our normal immune function state with T-cell mediated immunity. There are various ICI’s out in clinical practice and they target T cell receptors for CTLA-4, PD-1, PD-L1, and LAG-3 [2]. Examples of ICI’s include anti-CTLA-4 such as Ipilimumab and anti-PD-1 such as Pembrolizumab and Nivolumab. These drugs have been studied well in their impact to treat many cancers from melanoma to renal cell carcinoma [7–9] and the list of cancers susceptible to these medicines continues to grow exponentially.

## Cardiac Immune-related Adverse Events (irAEs)

### Introduction

We have learned more about the epidemiology, mechanism, characteristics, and long-term impact of ICI therapy on irAEs and cardiac irAEs more specifically over the past few years. The therapy promotes inflammatory and auto-immune effects from its downstream impact which clinically presents as irAEs. The mechanism likely roots from the pro-activation state of T cells when bound by the ICI therapy. This activation produces more pro-inflammatory cytokines. The surplus of these cytokines can cause the process known as cytokine release syndrome which is a non-specific inflammatory state that can impact a variety of body systems (10–11 causing inflammatory pathologies in organs such as the lung with pneumonitis, the gastrointestinal tract with colitis, the thyroid with thyroiditis. and the skin with dermatitis [8, 10].

Some of the most major and fatal adverse effects of ICI therapy have been seen in the cardiovascular system. The pro-inflammatory cytokines released after initiation of ICI therapy can lead to more free radical production which is especially cytotoxic to cardiac myocytes leading to downstream effects such as with conduction abnormalities, arrhythmias, and dysfunction in contractility [11, 12]. Some cardiac adverse effects include myocarditis, pericarditis, atrial fibrillation, myocardial infarction, cardiac arrest, Takotsubo cardiomyopathy, and cardiac failure. The WHO database mentions the most common cardiac irAEs are myocarditis and arrhythmias [2].

Several advancements in our understanding of cardiac irAEs have changed even just in the past few years. Part of this research has shown that myocarditis is not the only toxicity of ICI therapy however there truly is a broad variety of adverse cardiac events that can occur extending even into venous thromboembolic disease and accelerated atherosclerosis [13–15]. The understanding of the mechanism of ICI-induced toxicity has progressed considerably including the role of CD4 + and CD8 + T cells, immune homeostasis dysfunction, and promotion of inflammatory cytokines as a downstream product after ICI therapy [16, 17]. Retrospective studies in the past few years have found significance in serum markers such as elevation in creatinine kinase MB fraction (CK-MB), circulating troponin I (cTnI), and neutrophil-to-lymphocyte ratios (NLR) as clinical predictors of ICI-induced myocarditis. In addition, similar studies reported that the incidence of major adverse cardiac events was higher in cancer patients treated with ICI therapy over cancer patients treated with non-ICI therapy [18, 19]. These findings open future discussion on the importance of establishing standardized practice to monitor patients on ICI therapy with the inclusion of said cardiac biomarkers to help predict risk of adverse events. There is further need for monitoring these patients closely, which from the cardiac perspective could include routine biomarkers, electrocardiogram, and echocardiography. This is also recommended by the Heart Failure Association of the European Society of Cardiology which should be translated to practice moving forward given the high risk of serious cardiac events when they occur and high risk of mortality [15]. Cardiac specific adverse events have been seen to occur within the months to years range after initiation of therapy [13].

### Epidemiology

The epidemiology of cardiac irAEs has been better understood lately but obviously varies depending on the specific ICI or combination therapy used [2]. IrAEs can occur in about two-thirds of people and can lead to treatment termination in 40% of patients started on ICI [1, 2]. The incidence of cardiotoxicity within all types of irAEs is approximately 1%. The most prevalent cardiac irAE is myocarditis which is inflammation of the heart muscle tissue itself. Incidence of myocarditis from ICI therapy is about 0.04% to 1.14% [2]. The mortality rate from ICI therapy induced myocarditis is around 50% [1]. The median time to myocarditis diagnosis is about 65 days but can occur as early as two weeks after initiation of therapy. This draws attention to how serious watching for this adverse event should be given the high mortality rate, especially in a short period interval.

The Naqash et al. study reported anti-PD-L1 therapies had 0.6% MACE with myocarditis as the most common event [20, 21]. From another large database analysis, incidence rates for cardiac events per 10,000 person-years were reported as 3.4 for myocarditis, 142.3 for pericarditis, 10.3 for Takotsubo Cardiomyopathy, 17.2 for atrioventricular block, 1191.2 for heart failure, 55.2 for myocardial infarction, and 278.5 for stroke. Overall, the same study reported incidence of cardiovascular events was highest in the first 180 days after start of therapy [22].

Incidence reports may be low, but are severe with conditions such as myocarditis and heart failure. This emphasizes the importance of proper management to monitor patients on ICI therapy closely to look out for early signs and symptoms before they become severe or fatal.

## Mechanism of ICI-mediated Cardiovascular Toxic Effects

The potential cardiotoxicity related to immune checkpoint inhibitors (ICI) is still a paradigm [23]. Post-mortem histological analyses in patients treated with ICI found to have myocarditis have revealed the significant increase of inflammatory cells, with a predominance of T-cell lymphocytes and macrophages but absent of B-cell [24, 25]. These histological findings are consistent with the ICI mechanism of action [26].

There are several postulates about the pathophysiology behind these irAEs. The most well-accepted mechanism is the “shared antigen” concept between cardiomyocytes and some tumor cells [25, 27]. Moreover, the up regulation of CD4 + /CD8 + T lymphocytes with subsequent direct binding to cardiac cell surface, or the indirect destruction of cardiac cells via circulating cytokines or autoantibodies are other proposed mechanisms [2, 28].

### The “Shared Epitope” Theory

Current available data has demonstrated similarity in T-cell receptors or epitopes between cardiac myocytes and some tumor cells [29, 30]. In addition, an early immune evasion of these cardiac cells similar to tumor cells due to “shared epitope” can potentially be disrupted after ICI therapy predisposing to early development of myocarditis, as described in some cases [25, 30].

Similarly, the proposed “second hit”, akin to viral-myocarditis mechanism, is where tumor cells might play a role in systemic stress with cardiac cells involvement is pivotal for the development of immune reactivity [31]. Michael et al. demonstrated that mouse models with transplanted tumors developed left ventricular (LV) dysfunction compared to mouse models without tumors who did not develop LV involvement despite ICI therapy initiation.

### Direct Cellular Destruction of Cardiac Cells

Several studies have identified the expression of PD-L1 in the myocardium [32, 33], and similar to APC and tumor cells, they can activate PD-1/PD-L1 pathways limiting T-cell response under physiological conditions [34]. Similarly, the CTLA-4 pathway is activated and regulated by these cells. In this scenario, blockage of PD-L1/PD-1 or CTLA-4 pathways generates an over-activation of T-cells disrupting the homeostasis in the heart. Current data have identified the expression of PD-L1 in the myocardium of patients with ICI related myocarditis [25, 35]. The expression of PD-1and CTLA-4 on cardiac myocytes are suspected to be cardioprotective, as shown in biomedical studies [36]. For instance, PD-1 knockout mice ha4ve been described to develop LV dysfunction and dilated cardiomyopathy [37].

Likewise, the downregulation of PD-1 on CD4 + T cells and downregulation of PD-L1 in myeloid dendritic cells in patients with atrial fibrillation compared to healthy controls suggests that the downregulation of these checkpoints promotes T cell function and may contribute to the development or continuation of arrhythmias such as atrial fibrillation thru the mechanism of increasing cytokines such as IL-2 and IFN gamma, while suppressing regulatory cytokines such as IL-10 [38], potentially explaining how ICI-related arrhythmia events may transpire.

### Indirect Cellular Destruction of Cardiac Cells

The activation of certain cells, e.g. T-cells by ICI, upregulates the production of certain pro-inflammatory cytokines leading to a cascade of non-specific inflammatory processes known as cytokine release syndrome (CRS) [11, 39]. Interleukin-6 (IL-6), one of the most involved in CRS, among others (TNF-alpha, IFN-gamma, nitric oxide (NO), nitric oxide species (NOS), and reactive oxygen species (ROS) have been implicated in the development of arrhythmias, ventricular dysfunction, and other cardiac abnormalities [11, 12]. As per VigiBase, the WHO database of spontaneously reported suspected adverse drug reactions, CRS incidence ranges from 0.05% to 0.14% for ICIs, with a higher incidence among anti-PD-1/PD-L1 combination therapy [39].

## Clinical Risk Factors for ICI-medicated Cardiac Toxicity

Challenges exist to identify patients with risk of developing ICI-mediated cardiac toxicity. Identification of intrinsic factors to patients such as comorbidities, age, sex, genetics among others as well as factors related to therapy are crucial to recognize and prevent this condition. Hence, the importance of development of a risk predictive model before starting ICI, an area of research currently under progress.

### Patient Related-risk Factors

A number of proposed patient-related risk factors for cardiac irAEs have been described [2]. Pre-existing cardiovascular risk factors such as smoking, hypertension, obesity, diabetes, and a sedentary lifestyle and a history of heart disease such as presence of existing coronary artery disease or heart failure are common among ICI-related myocarditis [24, 40]. In the Phase III Javelin Renal 101 trial of ICI, an elevated baseline troponin level had a higher risk of major cardiac irAEs compared to a lower baseline troponin level [41]. Similarly, underlying auto-immune disorders such as rheumatoid arthritis, lupus erythematosus, sarcoidosis, and genetic factors including gene polymorphisms of CTLA-4, PD-1, or PD-L1 may have an increased risk for ICI irAEs [24, 42].

Regarding age, it is still unclear if a correlation exists since ICI-associated myocarditis occurs in a broad age range [40, 43]. Likewise, BMI has generated conflicting data in retrospective studies [44]. In relation to gender, there is a possible predominance of irAEs in females but with unclear physiologic explanations at present [44].

### Therapy-related Risk Factors

Combined ICI therapy (e.g. CTLA-4 inhibitor combined with a PD-1 /PD-L1inhibitor) appears to be a predominant risk factor for cardiac-related irAEs [2, 25]. A recent nationwide US database found that the incidence of irAEs requiring hospital admission was 3.5% for all ICI monotherapy, with some differences between the specific target therapy utilized: 3.3% for patients treated with anti-PD-1 antibodies, 1.1% for patients treated with anti-PD-L1 antibodies, and 3.9% for patients receiving anti-CTLA-4 antibodies [45]. Nevertheless, there was more than a double increase of hospitalizations with combination ICI therapy, with an incidence of 7.3% [45]. Similarly, the odd ratio of cardiac arrhythmias with combination ICI therapy was higher compared to ICI monotherapy (OR: 3.9, CI: 1.08–14.06, *p* = 0.603) [46]. Moreover, WHO database reported a high mortality rate with ICI myocarditis, which was more frequent with combination PD-1/CTLA-4 blockade, but also was noted with ICI monotherapy [47].

Likewise, the use of other conventional chemotherapies / target therapies (e.g. anthracyclines, anti-erbB2 drugs, Raf and MEK inhibitors, and VEGF tyrosine kinase inhibitors) have cardiotoxicity effects that could potentially be amplified with the use of ICI [42, 48]. For instance, taxanes cause myocardial injury via a massive histamine release or effects of subcellular organelles; anthracyclines have harmful cellular effect via mitochondrial injury; and 5-fluorouracil can lead to vasospasm, platelet aggregation and thromboxane formation via its noxious effects on the endothelium [49]. In the same way, targeted therapies such as anti-erbB2, BRAF as well as MEK inhibitors are known to induce reversible decline of left ventricular ejection fraction and, in some cases, enhance the risk of myocardial infarction, atrial fibrillation, and QTc prolongation [50, 51].

The combined effect of radiotherapy with ICI therapy might also boost the irAEs including cardiotoxicities [52, 53]. Further, a dose-dependent effect for irAEs has been described with combined therapies [54–56].

## Clinical Presentation, Diagnosis and Treatment

### Overview of Clinical Manifestations (Fig. 2)

**Fig. 2:**
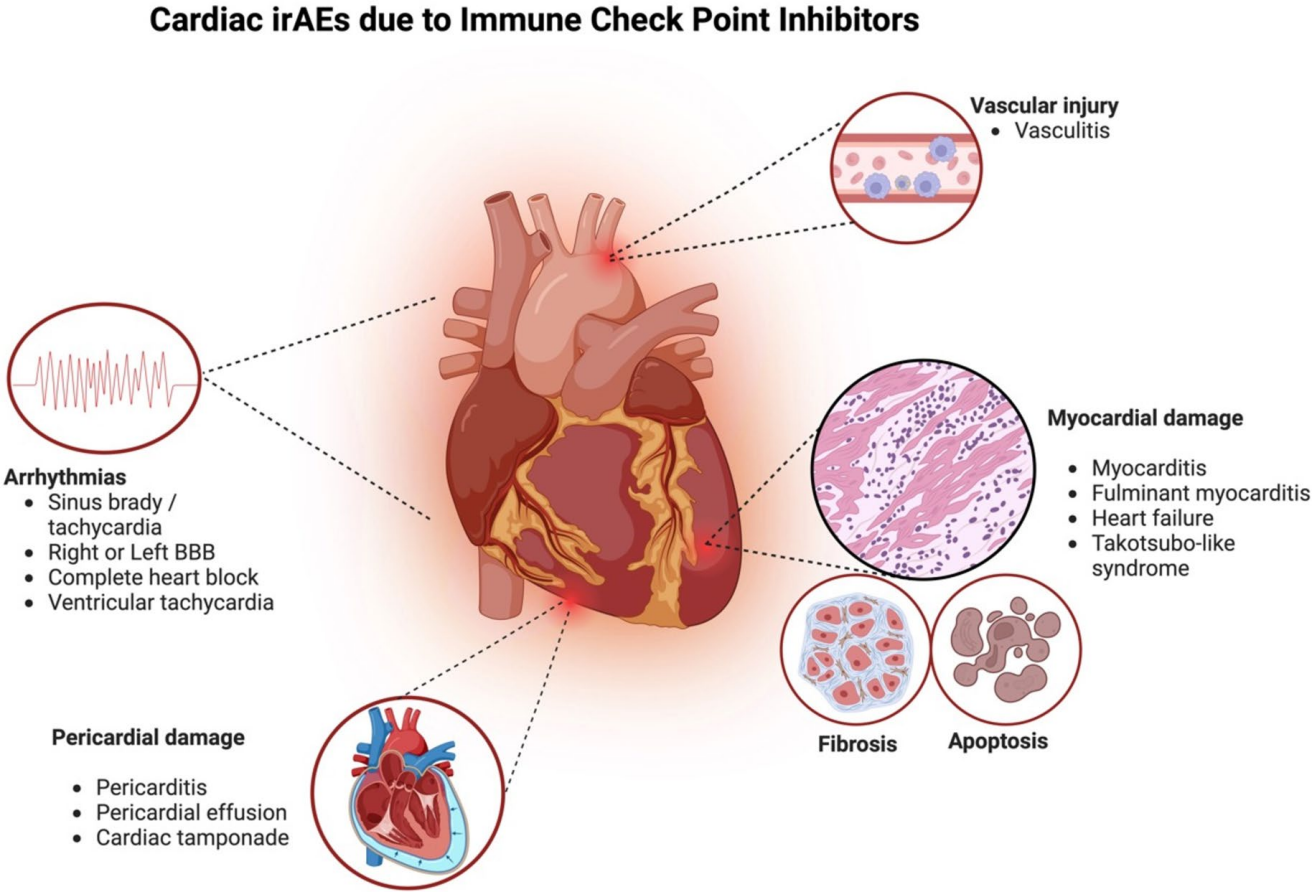
Clinical manifestations of cardiac irAEs due to ICIs. Key: Left BBB: left bundle branch block; irAEs: immune-related adverse events; ICIs: immune check point inhibitors

**Fig. 3:**
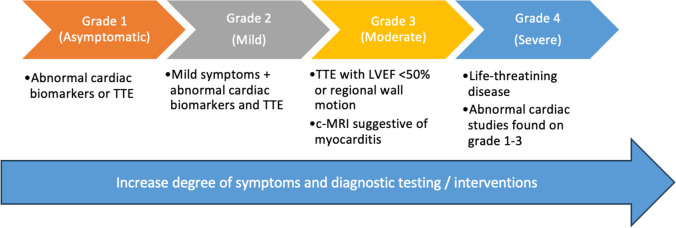
ASCO grading for ICI-induced myocarditis. Key: TTE: transthoracic echocardiogram; LVEF: left ventricular ejection fraction; c-MRI: cardiac magnetic resonance imaging

Cardiac irAEs present with a myriad of symptoms ranging from asymptomatic (with only abnormal elevations of cardiac biomarkers), to cardiac-related symptoms such as chest pain, dyspnea, palpitations, pre-syncope, syncope; and nonspecific symptoms such as fatigue or malaise. These constellations of symptoms create a broad differential which can be very easily mistaken or interpreted for other conditions including even non-cardiac irAEs (e.g. myositis, pneumonitis hypothyroidism) as well as related to the primary malignancy or comorbid conditions [23, 57]. In this scenario, the American Society of Clinical Oncology (ASCO) clinical practice guidelines for the management of irAE has classified cardiac irAEs based on the degree of symptoms ranging from asymptomatic irAE to severe irAE with end-organ damage [57] (Fig. 3). The most worrisome clinical picture is the severe end of this spectrum presenting as “fulminant” with an associated high mortality. In this scenario, patients present with cardiogenic shock, which might be accompanied by life-threatening arrhythmias such as advanced atrioventricular block or ventricular arrhythmias [25, 40, 58]. On the other hand, the moderate clinical spectrum can manifest as acute coronary syndrome-like [59], new onset of heart failure [60], or chronic heart failure [61]. In addition to the above-mentioned clinical syndromes, ICIs have been associated with pericardial effusions, thus, patients treated with ICIs can present with the clinical manifestations of these effusions [62].

The median timeline of cardiac irAE onset is about 3 to 9 weeks but can range from 2 to 54 weeks [63]. This period is related usually to after the first and third ICI infusion [43]. The time of presentation is particularly driven by the ICI therapy used, the cancer target, and the concomitant use of other therapies [62]. For example, anti-PD-L1 therapies had an earlier median onset of symptoms compared to anti-CTLA-4 therapies that present much later after initiation [63]. Herein, it is crucial to have a high index of suspicion of ICI-related cardiac irAEs to prevent potentially fatal outcomes.

### Diagnosis (Biomarkers, ECG, TTE, CMRI, and EMB) (Fig. 4)

**Fig. 4:**
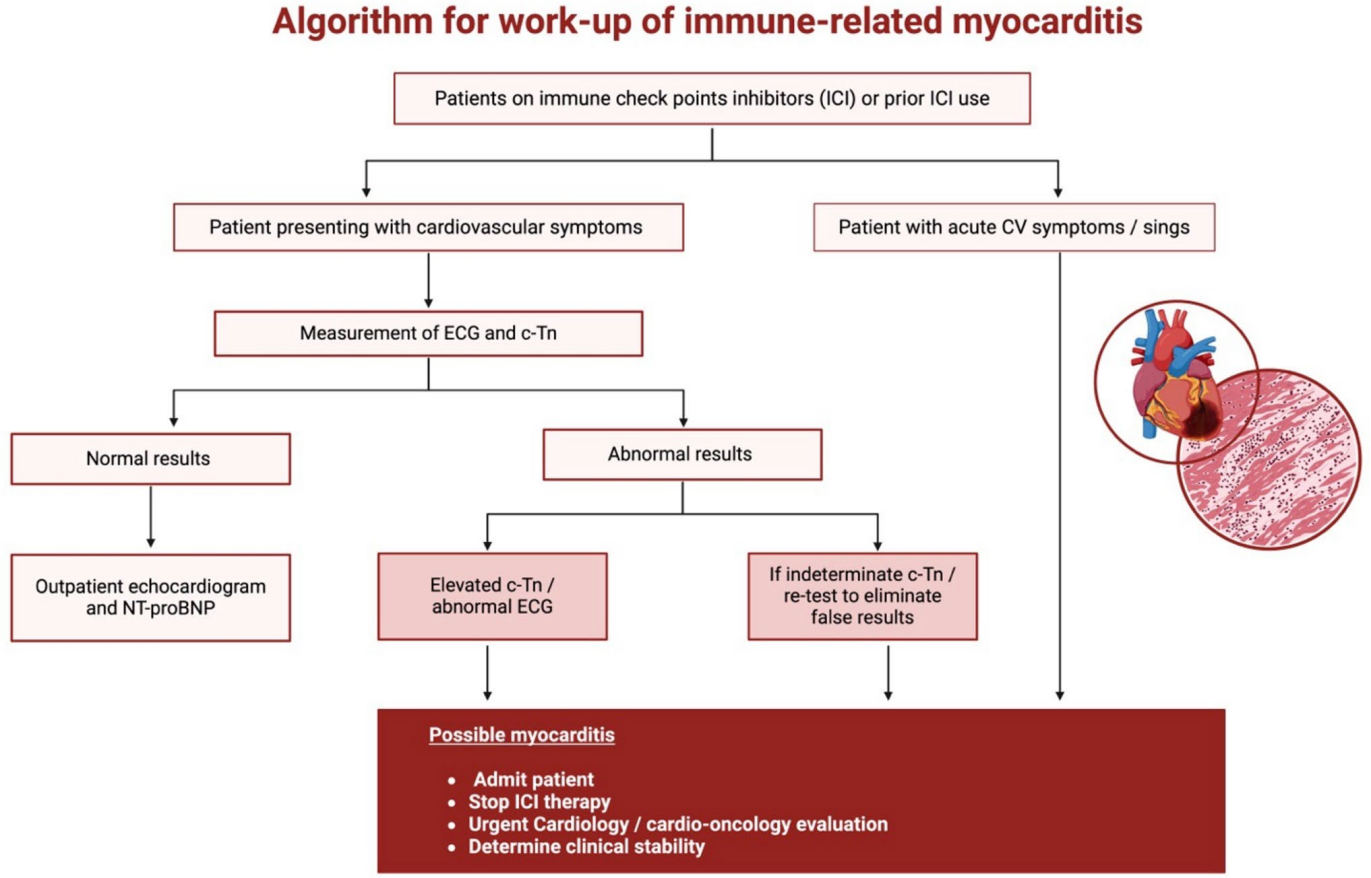
Algorithm for workup and management of cardiac irAEs due to ICI. Key: ECG: electrocardiogram; c-Tn: cardiac troponin; CV: cardiovascular, NT-proBNP: N-terminal pro b-type natriuretic peptide

#### Biomarkers

Specific cardiac biomarkers are needed to anticipate the development of cardiac irAEs from ICI therapy. However, there is still a lack of specific biomarkers that achieve this role. Thus, imputed biomarkers such as cardiac troponin (cTn) have been used as a surrogate of the development of irAEs in high-risk patients [64]. Both cardiac troponin T (cTnT) and cardiac troponin I (cTnI) are solely found in the myocardium and are elevated in the majority of the time in patients with cardiac irAEs [40, 43, 47]. Meanwhile, N-terminal pro brain-type natriuretic peptide (NT-proBNP) and brain-type natriuretic peptide (BNP) have been used as a hallmark in the diagnosis and management of heart failure, but with an unclear role in the risk analysis of ICI cardiotoxicity. A retrospective study showed an increased risk of irAEs in patients with BNP > 100 pg/ml [65].

#### Electrocardiogram

An electrocardiogram (ECG) should be obtained especially when presenting with symptoms of chest pain, dyspnea, or palpitations while on ICI therapy. Between 40–89% of patients with ICI related cardiac events have abnormal ECGs [66]. The most common ECG abnormality is nonspecific T waves changes, and other reported ECG abnormalities include changes in PR interval, ST depression or elevation, atrioventricular blocks, ventricular arrhythmias, and Q waves [57, 67]. Acquisition of a baseline ECG is imperative as a comparison to subsequent studies.

#### Cardiac Imaging

##### Echocardiography

Imaging is the next step for evaluation of cardiac irAEs, and transthoracic echocardiogram (TTE) is an essential tool. TTE provides information on the myocardium itself and if there are any new changes in ventricular function. Patients with a severe syndrome, such as fulminant myocarditis, will have abnormal ventricular function at presentation or regional wall motion abnormalities [40, 61]. In more subtle presentations, diastolic dysfunction with preserved ejection fraction can be identified [61]. Global longitudinal strain (GLS) is a post-processing technique used to assess left ventricular systolic function. In a study by Awadalla et al., GLS was shown to be abnormal in patients with ICI myocarditis, and when compared to controls, was lower among patients presenting either with preserved or reduced LVEF [68]. The development of a new pericardial effusion also may support the diagnosis of myocarditis. The ASCO clinical practice guidelines for prevention and monitoring of cardiac dysfunction in survivors of adult cancers as well as the European Society of Cardiology Guidelines of Cardio‐Oncology recommend obtaining a baseline echocardiogram before initiation of any potentially cardiotoxic therapy [69, 70]. There are no clear American guidelines regarding cardiac irAEs in patients with ICI therapy [71].

##### Cardiac Magnetic Resonance Imaging

Cardiac magnetic resonance imaging (c-MRI) is one of the most sensitive cardiovascular modalities to detect early signs of inflammation and fibrosis in the myocardium. Hence, it has the most robust validation to diagnose myocarditis noninvasively [72]. There are a variety of c-MRI modalities that have been validated to detect myocarditis such as T2‐weighted imaging [73, 74], late gadolinium enhancement (LGE) [75], extracellular volume fraction [76], T1 mapping [76, 77], and T2 mapping [76, 78]. The modified Lake Louise Criteria is currently used to define ICI-related myocarditis [79, 80]. In a case series by Mahmood et al., patients with the diagnosis of ICI associated myocarditis with c-MRI, mid-myocardial LGE was associated with major cardiovascular events, but LGE was only present in 26% of these patients [40]. Other studies showed septal LGE in only 48% of patients with cardiac irAEs [81]. The low sensitivity of c-MRI with LGE could be caused by early imaging or by the non-specific pattern of ICI-associated myocarditis, where the extracellular space might not be significantly affected as seen in other forms of myocarditis [82]. Further studies are needed to continue to elucidate the role and characteristic of c-MRI in this pathology. There is more needed to be determined on diagnostic and classification criteria using imaging modalities.

##### Endomyocardial Biopsy (Table 1)

**Table 1:** Endomyocardial biopsy grading

EMB grading by Palaskas et al. criteria [86]	
Grade	Pathologic features
0	Negative
1: Myocardial inflammation	Multifocal inflammatory infiltrated without overt cardiomyocytes loss by light microscopy
1A	Mild inflammatory cell score (10–20 inflammatory cells/high power field
1B	At least moderate inflammatory cell score (> 20 inflammatory cells/high power field)
2: Definite myocarditis	Multifocal inflammatory cell infiltrates (> 40 inflammatory cells /high power field)
EMB by Champion and Stone criteria [87]	
Grade	Immunohistochemistry
Low grade	50 CD3 + cells/high power field
High grade	> 50 CD3 + cells/high power field

*EMB* endomyocardial biopsy

The gold standard for diagnosis of ICI-related myocarditis is endomyocardial biopsy (EMB). This step should be considered if concerns arise from a combination of clinical symptoms of myocarditis along with imaging corresponding to such with patients on ICI therapy, severe syndrome, or non-responders to initial therapy with steroids.

The features on the EMB pathognomonic for ICI-related myocarditis include inflammatory changes with interstitial fibrosis along with infiltration of T-cells (CD-4, CD-8), macrophages, and other inflammatory changes [83]. Over more than two decades, the Dallas criteria was proposed and implemented for histopathological categorization of the diagnosis of myocarditis [84]. However, the need of an inflammatory infiltrate associated with myocyte necrosis or damage not characteristic of an ischemic event, creates a challenging diagnosis in subtle myocarditis cases. Moreover, sampling errors, variability within pathologist’s interpretation, inconsistency of markers of viral infection with immune activation, and discrepancy of treatment outcomes advocate that the Dallas criteria is outdated [85].

Recently, Palaskas et al. developed a system to classify ICI-related myocarditis based on the pathology findings and noted a correlation with clinical outcomes [86]. Similarly, Champion and Stone used the number of inflammatory cells in the myocardium to grade ICI-related myocarditis, where high-grade patients had a severe clinical course [87].

Understanding the advantages and limitations of EMB and complications is crucial to define the appropriate and adequate timing use of this invasive diagnostic tool.

### Treatment

Medical management of cardiac irAEs is a multi-team approach between the cardiologist and oncologist.

The fundamental treatment for ICI related cardiotoxicity is corticosteroids including oral prednisone or intravenous methylprednisolone [40, 71]. In the same manner, there should be a low threshold to initiate corticosteroids if there are concerns for cardiac irAEs. The challenge lies in identifying the people who would benefit the most from starting therapy, considering there is a transient or permanent hold of ICI therapy, which could be detrimental for the underlying oncologic disease. As per current ASCO clinical guidelines, in Grade 1 cardiac irAEs, temporary hold of ICIs is recommended. However, in Grade 2 or above, discontinuing ICIs permanently is advocated [69]. Further, all patients require initiation of corticosteroids at 1–2 mg/kg daily independently of the grade of ICI-related cardiac toxicity [69].

In non-responder cases, high-dose intravenous corticosteroids (1gr daily) should be considered. Addition of adjuvant therapy with intravenous immunoglobulins [88], mycophenolate [88], infliximab [89], anti-thymocyte globulin [90], plasmapheresis [89], alemtuzumab [91], and abatacept [92] have been successful in some case reports, but their effectiveness is still unclear. Hence, their use is limited for patients with no immediate response and manifesting severe signs of cardiovascular toxicity (shock, recalcitrant ventricular arrhythmias).

There is no clear consensus on the duration and how to taper the corticosteroids. The ASCO guidelines recommend a taper of at least 4–6 weeks, a shorter period compared to viral myocarditis trials, where corticosteroids were continued for at least 3 months to 1 year [61]. In ICI-related myocarditis, the dose and duration of corticosteroids vary depending on the clinical improvement, and where c-Tn levels are essential for monitoring.

Also, patients should be treated with established therapy depending on their clinical syndromes. For instance, in acute decompensated heart failure, diuretics, along with inotropes and mechanical circulatory support (if indicated) are crucial [93]. Arrhythmias including ventricular arrhythmias and brady-arrhythmias are managed based on conventional guidelines [68, 94, 95].

## Conclusion

Cardiac irAEs related to ICI therapy is an uncommon but complex medical condition with a broad clinical presentation, challenging diagnosis, and anecdotal medical therapy. For this reason, a high index of suspicion is crucial to prevent deadly outcomes. Both the oncologist and the cardiologist need to be vigilant for the development of symptoms in patients, particularly the ones with higher risks. An early clinical and multimodality evaluation is paramount to diagnose and treat this population. State-of-art therapy is based on corticosteroids, with a potential role of immunomodulators in non-responder cases. Clinical studies are needed to elucidate further mechanisms and diagnostic strategies as well as to establish clear guidelines for treatment of these cardiac irAEs due to ICIs.

## Key References

- Moradi A, Kodali A, Okoye C, et al. A Systematic Review of Myocarditis Induced by Immune Checkpoint Inhibitors: How Concerning Is the Most Common Cardiotoxicity of Immune Checkpoint Inhibitors?. Cureus (2023) 15(7): e42071.
  - ∘ This systematic review from 2018 to 2020 describes the most common cardiac irAEs related to ICIs. It emphasizes that even if it is low and usually mild or asymptomatic, there are deadly cardiac irAEs that physicians need to be aware of.
- Tocchetti CG, Farmakis D, Koop Y, Andres MS, Couch LS, Formisano L, et al. Cardiovascular toxicities of immune therapies for cancer—a scientific statement of the Heart Failure Association (HFA) of the ESC and the ESC Council of Cardio-Oncology. Eur J Heart Fail. 2024 Aug 1.
  - ∘ In this European scientific statement, they describe broadly the epidemiology, mechanism, diagnosis and management of cardiac-irAEs of the immune-checkpoints inhibitors. This sets the precedent for future guidelines.

## Data Availability

No datasets were generated or analysed during the current study.
